# Exploring the responsiveness of goal attainment scaling in relation to number of goals set in a sample of hemophilia-A patients

**DOI:** 10.1186/s41687-019-0110-9

**Published:** 2019-04-01

**Authors:** Lisa McGarrigle, Jonathan C. Roberts, Michael Denne, Kenneth Rockwood

**Affiliations:** 1 Division of Geriatric Medicine, Department of Medicine, Dalhousie University and Nova Scotia Health Authority, 5955 Veterans’ Memorial Lane, Halifax, NS B3H 2E1 Canada; 2 DGI Clinical Inc, 1730 Market St, Halifax, NS B3J 3N9 Canada; 3 Bleeding & Clotting Disorders Institute, Peoria, IL USA; 4 Shire, US Medical Affairs, Chicago, IL USA; 5 Centre for Health Care of the Elderly, Nova Scotia Health Authority, 1421-5955 Veterans’ Memorial Lane, Halifax, Nova Scotia B3H 2E9 Canada

**Keywords:** Patient, Goal attainment, Responsiveness, Hemophilia

## Abstract

**Purpose:**

Guidelines for the use of goal attainment scaling (GAS) recommend that the patient specify at least three goals. Even so, this may not always be feasible or align with patient preferences. Investigations into the psychometric properties of GAS using three or more goals largely support its reliability, validity, and responsiveness compared with standard measures. As evaluations of responsiveness rely on variability estimates, this metric may be impacted when GAS is based on fewer than three goals. For this reason, we investigated the responsiveness of one- and two-goal GAS.

**Methods:**

Secondary analyses were conducted on data from a mixed sample of pediatric, adolescent and adult subjects with hemophilia A. The standardized response mean (SRM) and its 95% confidence intervals (CI) were used to assess responsiveness of one- and two-goal GAS at six and twelve weeks.

**Results:**

Both one-goal and two-goal GAS demonstrated similar responsiveness to change at 6-week (Patient-Rated GAS: one-goal SRM [95% CI] = 0.70 [0.45–1.08], two-goal = 0.96 [0.68–1.30]; Clinician-Rated GAS: one-goal = 1.26 [0.81–1.77], two-goal = 1.01 [0.73–1.32]) and 12-week follow-up (Patient-Rated GAS: one-goal SRM [95% CI] = 1.14 [0.53–1.71], two-goal = 1.35 [0.92–1.82]; Clinician-Rated GAS: one-goal = 1.71 [1.12–2.30], two-goal = 1.48 [1.02–2.02]). Larger SRMs were observed for clinician-rated GAS, but all were within the rubric of a large effect size.

**Conclusions:**

One-goal GAS is responsive to change in a clinical population. Further research is recommended in a larger sample where responsiveness of one- and multiple-goal GAS can be compared

## Introduction

In the evaluation of clinical meaningfulness, individualized outcome measures, paired with standardized assessments, allow valid evaluations that combine reproducibility and inherent patient -centeredness. Individualization self-consciously aims to incorporate patient preferences, an important step in understanding clinical meaningfulness [1]. Well-developed patient-reported outcome measures provide essential information to enhance patient healthcare and quality of life [2]. Sometimes, however, what patients prefer might not extend to methods by which their preferences are elucidated. We have encountered such an example in relation to goal attainment scaling (GAS). GAS [3] allows the patient, usually in consultation with their clinician, to set personalized treatment goals. In contrast with psychometric measures which specify domains or symptoms to be evaluated, GAS allows people to select goals according to their individual needs, thus maximizing the relevance to the patient and the non-arbitrariness of symptom measurement. GAS can be viewed as a standardized approach to the measurement of change in response to patient -centered and solution-focused care. Goal-setting is usually conducted in consultation with a clinician, and so can facilitate shared decision-making about treatment. The key feature of GAS is its ability to detect clinically meaningful change due to its focus on outcomes that are important to the individual [4].

The extent of goal attainment is rated on a 5-point scale ranging from + 2 (much better than expected) to − 2 (much worse than expected). Baseline status is generally set at − 1 and a follow-up attainment score at this level is considered somewhat worse than expected. The expected outcome, or “goal”, is set as 0. GAS is frequently used to track symptom changes due to a therapeutic intervention, and often operates as a co-intervention; formal goal-setting inherent in GAS may in itself encourage progress in specified goal areas [5]. This can be especially useful in dementia [6] and traumatic brain injury [7] in which goal-setting ability is compromised.

GAS guidelines recommend that patients set at least three goals, generally weighted in order of importance to the patient [8]. In practice this might not always be feasible – or appropriate, especially when it does not accord with patient preferences. When GAS is being used with an intervention designed to address a range of problems, it is likely the number of goals specified will be multiple. In observational/non-interventional studies that capture change over the course of usual care, patients might wish to set only one or two goals based on the symptoms most relevant to them.

Investigations of the reliability, validity, and responsiveness of three-goal GAS have supported the psychometric properties of the tool, particularly its responsiveness compared with standard measures [9, 10]. The psychometric properties of one-goal GAS have not yet been investigated. It is likely that having only one goal will have the most impact on measures where variability is key, e.g., effect size. The number of goals a patient specifies will affect the GAS formula which is calculated as follows:

$$ T=50+\frac{10\sum {w}_i{x}_i}{\sqrt{\left(1-\rho \right)\sum {w}_i^2+\rho {\left(\sum {w}_i\right)}^2}} $$

where T is the composite score, *w*_i_ is the weight assigned to the *i*th goal, *x*_i_ is the numerical value (− 2 to + 2) of the attainment level of the *i*th goal, and *ρ* is the estimated correlation between goal scores (assumed to be constant and set at .3) [3]. With one goal, the scale can only produce one of five distinct values (− 2 to + 2), whereas with three goals the sum of the scale scores will have a range of thirteen distinct values (− 6 to + 6), so that a summary GAS score will have a 13-point scale [8]. Briefly, the consequence of having only one goal is that responsiveness may be particularly impacted when GAS is based on one goal as evaluations of responsiveness rely on variability estimates. Our objective was to conduct a first investigation into the responsiveness of one-goal GAS.

## Methods

### Participants

Secondary analyses were conducted on data from the standardized Goal Attainment Scaling menu for Hemophilia (GAS-Hēm) study, a 12-week, prospective study of the feasibility of using a standardized GAS menu to track clinical change in a mixed sample of pediatric, adolescent and adult subjects with hemophilia A [11]. Participants were recruited through four clinical sites in the USA (*n* = 2) and Canada (n = 2) between December 2015 and August 2016. Participants were eligible if they were aged between 5 and 65 years, had a diagnosis of hemophilia A with clotting factor levels of 5% or less, were on a prescribed regimen of continuous prophylaxis, and were proficient in English. Forty-four participants and/or their legal guardians provided informed consent and were screened for study participation. Two participants were excluded (one did not meet the eligibility criteria; one did not return for the baseline visit). A further two participants were excluded as they did not provide follow-up data. Of 40 participants included in the analysis sample, 7 were pediatrics (aged 5–12 years), 9 were adolescents (13–18 years), and 24 were adults (19–65 years). As expected - hemophilia A is very rare in females - all participants save one were male.

### GAS-Hēm

GAS-Hēm is an online instrument developed with input from hemophilia A patients and expert practitioners. It consists of 29 goal areas that reflect challenges frequently encountered by people with hemophilia A. Participants had the option to define their own goals, with 59% opting to do so. GAS involves formal setting of appropriate goal attainment levels, which can prove challenging and time-consuming to healthcare professionals. GAS-Hēm was designed to facilitate the goal-setting process using Web-enabled software initially developed by our group for use in dementia [12]. Findings from the GAS-Hēm feasibility study suggest that GAS-Hēm can enhance patient-centered care and compliment standard measures of clinically meaningful change [11]. At baseline, participants underwent a clinician-facilitated goal-setting session using the GAS-Hem tool. Follow-up assessments were conducted at six and 12 weeks. Participants were encouraged to set three goals, however, half chose to set one goal, and half set two. When participants set two goals, goals were ranked in order of importance. Clinicians also scored participants’ goal attainment at follow-up visits.

### Data analysis

With only one goal, there is no intercorrelation between goals, and no weighting, so the GAS formula simplifies as follows:

$$ GAS=50+(10x) $$

where *x* is the numerical value (− 2 to + 2) of the attainment level. This impacts the estimation of responsiveness, in which the variability of scores is key. We estimated responsiveness using standardized response means (SRMs) [13]. SRMs were calculated for one- and two-goal GAS at six and 12 weeks by dividing the mean change (follow-up minus baseline) by the standard deviation of the change. SRMs are considered to be a relatively unbiased measure of effect size [14] and were interpreted using Cohen’s effect size criteria, where a value of 0.2 was considered “small”, 0.5 “medium”, and 0.8 “large” [15]. Independent t-tests were conducted to compare change scores for one- and two-goal GAS. SRMs and their bootstrap confidence intervals (CIs) were calculated using MedCalc for Windows, version 18.6 [16].

## Results

### Sample characteristics

Half the patients were aged 23 years or less, and most were adults. The people who set one GAS goal most often were children or adolescents (Table 1).

**Table 1:** Sample characteristics at baseline

	Age Group			
	Pediatric	Adolescent	Adult	All Subjects
Age				
n	7	9	24	40
Mean (sd)	8 (2)	15 (2)	33 (12)	24 (14)
Min-Max	5–11	13–18	19–64	5–64
Sex: n (%)				
Male	7 (100)	8 (88.9)	24 (100)	39 (97.5)
Female	–	1 (11.1)	–	1 (2.5)
Education Level: n (%)				
Elementary/Grade School	5 (71.4)	3 (33.3)	–	8 (20)
Some High School	–	6 (66.7)	1 (4.2)	7 (17.5)
High School Graduate	–	–	6 (25)	6 (15)
Some College/University	–	–	6 (25)	6 (15)
College/University Graduate	–	–	7 (29.2)	7 (17.5)
Graduate Degree	–	–	4 (16.7)	4 (10)
Other	2 (28.6)	–	–	2 (5)
Number of Goals: n (%)				
Subjects with one goal	5 (71.4)	7 (77.8)	8 (33.3)	20 (50)
Subjects with two goals	2 (28.6)	2 (22.2)	16 (66.7)	20 (50)

### Responsiveness results

Baseline and follow-up GAS scores and SRMs are presented in Table 2. Patient-rated one-goal and two-goal GAS demonstrated similar responsiveness to change at 6-week (one-goal SRM [95% CI] = 0.70 [0.45–1.08]; two-goal SRM [95% CI] = 0.96 [0.68–1.30]) and 12-week follow-up (one-goal SRM [95%CI] = 1.14 [0.53–1.71]; two-goal SRM [95% CI] = 1.35 [0.92–1.82]). There were no significant differences in mean change scores between patient-rated one- and two-goal GAS at 6-weeks (mean difference = 3.7, *p* > .05) and 12-weeks (mean difference = 4.7, p > .05). For clinicians, SRMs for one-goal and two-goal GAS were of similar magnitude at 6-week (one-goal SRM [95% CI] = 1.26 [0.81–1.77]; two-goal SRM [95% CI] = 1.01 [0.73–1.32]) and 12-week follow-up (one-goal SRM [95% CI] = 1.71 [1.12–2.30]; two-goal SRM [95% CI] = 1.48 [1.02–2.02]). There were no significant differences in mean change scores between clinician-rated one- and two-goal GAS at 6-weeks (absolute difference = 1.1, *p* > .05) and 12 weeks (absolute difference = 3.8, p > .05).

**Table 2:** Patient and clinician GAS scores and SRM for one- and two-goal GAS

	Baseline	6-Weeks	12-Weeks
One-Goal GAS (m, sd)			
Patient	40.0 (0.0)	46.5 (9.3)	52.0 (10.6)
Clinician	40.0 (0.0)	50.0 (7.9)	54.0 (8.2)
Two-Goal GAS (m, sd)			
Patient	37.6 (0.0)	47.8 (10.7)	54.3 (12.4)
Clinician	37.6 (0.0)	48.7 (11.0)	55.4 (12.1)
One Goal Responsiveness (SRM)			
Patient	–	0.70	1.14
Clinician	–	1.26	1.71
Two Goal Responsiveness (SRM)			
Patient	–	0.96	1.35
Clinician	–	1.01	1.48

## Discussion

SRM values at 6- and 12-week follow-up support the responsiveness of one-goal GAS in a sample of participants with hemophilia A. In participants who set only one goal, SRMs at 6- and 12-weeks were moderate to large. For clinician-rated one-goal GAS, SRMs at both time-points were large. A similar trend was observed for two-goal GAS, and all effect sizes can be considered large according to Cohen’s criteria. This suggests that GAS is responsive, even when participants set only one goal. The slightly larger SRMs for two-goal patient-rated GAS may be due to larger mean differences in scores. Although mean change was not significantly different for one- and two-goal GAS, it is possible that an increased number of goals provides greater opportunity to detect change. Even so, the opposite trend was observed for clinician-rated GAS with slightly larger SRMs for one-goal compared to two-goal GAS. Overall, findings support previous studies where large SRMs were observed for GAS [17, 18].

Establishing the responsiveness of one-goal GAS has implications for the use of this tool in that even greater individualization can be achieved if participants are not restricted by a minimum goal number. Here, participants were encouraged to set three goals, however in practice this did not occur. This may reflect the fact that GAS-Hēm was non-interventional and not designed to target numerous problem areas, unlike many interventional studies that have incorporated GAS [9, 17, 19].

The lower number of goals set may also be due to the unique aspects of hemophilia A and its impact on patients’ lives. Hemophilia A is a genetic disorder characterized by deficiency in coagulation factor VIII (FVIII), and modern therapy revolves around prophylactic FVIII replacement [20]. In understanding clinical meaningfulness for this patient population, goals tended to encompass optimizing prophylaxis to positively impact goal areas such as joint problems, leisure activities, being able to administer factor, weight, exercise and nutrition. In this sense, improved adherence acts as a means to an end, and from the perspective of many people living with hemophilia, can be viewed as a summative measure that integrates a range of outcomes. If individuals with hemophilia are not adherent to prophylaxis, they will be at increased risk for bleeding events with their proposed physical or leisure activities and unlikely to achieve their goal. Further, some individuals with hemophilia who have successfully integrated prophylaxis into their routine have stated it is a prerequisite for “normal life” [21]. As nonadherence to prophylaxis therapy is associated with worse outcomes in hemophilia [22] it is not surprising that improving this aspect of hemophilia patients’ lives may be important to them [21].

The small sample size in this study limits the generalizability of findings and results are suggestive. GAS-Hēm was also limited as comparative responsiveness with the recommended three or more goals was not possible. Additionally, the fact that younger participants tended to set one goal presents a potential confound as it is unclear whether the responsiveness of one-goal GAS is influenced by age. Of those who set one goal, 60% (12/20) were children/adolescents, whereas only 20% (4/20) of those who set two goals were children/adolescents. As such, groups which set one vs. two goals are somewhat heterogenous, rendering comparisons of SRMs problematic. Even so, one of the major advantages of GAS is its ability to produce standardized scores reflecting the degree of goal attainment across a range of disease areas and ability levels [8]. Given the individualized nature of GAS, not everyone is expected to set goals in the same areas nor to have the same baseline description for similar goals. Rather, the key inference in GAS is the extent to which participants attained their own personal goals at follow -up. Here, goal-setting was facilitated by a trained professional to ensure that goals were age-appropriate and sufficiently challenging for all participants and a graded series of likely outcomes was constructed in a systematic way (the 5-point goal attainment scale). This may have contributed towards minimizing any potential age confounds, but even so, the impact of age on GAS responsiveness can only be firmly established through further research in homogenous groups. Future research should also investigate inter-rater reliability and/or validity of one-goal GAS in relation to comparative measures targeting changes or improvement. Additionally, the generalizability of findings across disease areas, such as dementia, is unclear. Our findings raise interesting questions which need to be explored across disease areas and in larger samples in which more goals have been set.

## Conclusion

These preliminary findings highlight the potential for further individualization of GAS by allowing participants to select fewer goals than are currently imposed by standard GAS guidelines. SRMs suggest that one-goal GAS is responsive to change in a clinical population. Further research is recommended in a larger sample where responsiveness of one- and multiple-goal GAS can be compared.

## Abbreviations

CI: Confidence Interval
GAS: Goal Attainment Scaling
GAS-Hēm: Goal Attainment Scaling menu for Hemophilia
SRM: Standardized Response Mean
